# The overlooked challenges facing out-of-hours primary care in the NHS: a missed opportunity in policy

**DOI:** 10.3399/BJGPO.2024.0292

**Published:** 2025-03-26

**Authors:** Alexandra L Creavin, Sam T Creavin

**Affiliations:** 1 Bristol Medical School, University of Bristol, Bristol, UK

**Keywords:** NHS, Policy, Primary Healthcare, Inequality

## Introduction

Out-of-hours (OOH) primary care is a crucial health service for patients needing attention outside regular office hours, yet it was largely overlooked in Lord Darzi’s recent landmark healthcare review.^1^ This gap leaves OOH services underrepresented in both policy discussions and research. Integrating OOH services more fully into policy frameworks like the Darzi report would not only elevate their importance but also support reforms essential to sustaining this vital NHS function.

Our recent engagement with GPs, allied health professionals, commissioners, and patients revealed six key challenges within OOH primary care that, if addressed, could strengthen healthcare access, efficiency, and patient outcomes. Here, we explore each challenge and suggest ways to help preserve OOH care as a crucial NHS component.

### Disconnect between in-hours and out-of-hours care

A major issue for OOH services is limited integration with in-hours care. Without a shared electronic health record and a unified handover system, patients experience disjointed care journeys, being passed from one service to another without resolution. This lack of continuity leads to delays, repeat consultations, and a fragmented patient experience.

**Recommendation:** Establish a secure electronic health record accessible across in-hours and OOH services to improve patient transitions and reduce unnecessary visits.

### Growing demand on OOH services

As in-hours GP access becomes increasingly limited, OOH services have seen a rise in non-urgent cases, stretching already limited resources. The demand for palliative and end-of-life care is also growing, requiring GPs to navigate complex family conversations and manage intricate care plans during OOH hours.^2^ Additionally, mental health support is a major and growing need within OOH, adding further complexity.

**Recommendation**: Explore novel interventions for managing palliative care and mental health needs outside office hours, such as direct access to community specialists.

### Workforce struggles and the loss of experienced GPs

OOH services have traditionally relied on experienced GPs, but this dynamic is shifting as more senior GPs approach retirement. Fewer newly qualified GPs are exposed to OOH work during training, which can limit their confidence and willingness to work in unscheduled care settings. Stakeholders noted a reduction in consultation rates and the lack of experienced GPs to model efficient, safe practices in these high-stakes environments.

**Recommendation**: Create dedicated OOH training modules for trainee GPs to ensure preparedness and encourage experienced GPs to serve as mentors for newer practitioners.

### Economic disincentives

Financial disincentives are significant deterrents for GPs working OOH shifts. Pay stagnation, tax penalties, and pension-related issues have lowered take-home earnings, leading to reluctance among GPs to take OOH shifts.

**Recommendation:** Adjust pay and tax structures to improve OOH shift attractiveness and support GPs willing to provide essential after-hours care.

### Limited access to medications

OOH providers often struggle with limited access to essential medications, as a lack of extended-hour pharmacies restricts patient care.^3^ While some OOH services hold on-site medications, this is not always sufficient, and some patients still rely on emergency departments for prescriptions, which delays care and strains resources.

**Recommendation**: Increase access to essential medications OOH including through 24-hour pharmacies and exploration of novel interventions.

### Inequities in access and care quality

Barriers to OOH care disproportionately affect vulnerable populations, exacerbating health inequalities.^4^ Patients with learning difficulties, with dementia, or without English as a first language may find remote consultations challenging, and residents in rural areas face limited transportation options, making it difficult to access OOH appointments.^5–8^ Individuals with multimorbidity are especially impacted by these barriers, experiencing compounded issues when trying to obtain timely OOH care.

**Recommendation**: Improve data collected regarding barriers to access and patient care pathways to improve equitable access to OOH care.

### Data gaps and underrepresentation in research

The fragmented nature of OOH providers complicates efforts to gather comprehensive data on service dynamics, care quality, and patient outcomes. This lack of routine freely available data may partly explain why OOH care was underrepresented in the Darzi report, limiting insights into its challenges and hindering the generation of evidence-based solutions.^2^

**Recommendation**: Encourage standardised data sharing across OOH services to support future research and provide policymakers with the evidence needed to address these critical gaps.

## Conclusion

Like the rest of the NHS, OOH services stand at a critical juncture, facing multifaceted challenges that need immediate policy attention. By incorporating OOH care into major policy reform, policymakers could develop a roadmap to support this essential service. Addressing workforce shortages, improving national access to data, and fostering integration with in-hours care are crucial steps forward. The health of our most vulnerable populations depends on it.
